# The Impact of Anterior Thalamic Lesions on Active and Passive Spatial Learning in Stimulus Controlled Environments: Geometric Cues and Pattern Arrangement

**DOI:** 10.1037/a0036280

**Published:** 2014-04

**Authors:** Julie R. Dumont, Nicholas F. Wright, John M. Pearce, John P. Aggleton

**Affiliations:** 1 School of Psychology, Cardiff University, Cardiff, United Kingdom

**Keywords:** cognitive map, geometry, latent learning, navigation, place learning, thalamus

## Abstract

The anterior thalamic nuclei are vital for many spatial tasks. To determine more precisely their role, the present study modified the conventional Morris watermaze task. In each of 3 experiments, rats were repeatedly placed on a submerged platform in 1 corner (the ‘correct’ corner) of either a rectangular pool (Experiment 1) or a square pool with walls of different appearances (Experiments 2 and 3). The rats were then released into the pool for a first test trial in the absence of the platform. In Experiment 1, normal rats distinguished the 2 sets of corners in the rectangular pool by their geometric properties, preferring the correct corner and its diagonally opposite partner. Anterior thalamic lesions severely impaired this discrimination. In Experiments 2 and 3, normal rats typically swam directly to the correct corner of the square pool on the first test trial. Rats with anterior thalamic lesions, however, often failed to initially select the correct corner, taking more time to reach that location. Nevertheless, the lesioned rats still showed a subsequent preference for the correct corner. The same lesioned rats also showed no deficits in Experiments 2 and 3 when subsequently trained to swim to the correct corner over repeated trials. The findings show how the anterior thalamic nuclei contribute to multiple aspects of spatial processing. These thalamic nuclei may be required to distinguish relative dimensions (Experiment 1) as well as translate the appearance of spatial cues when viewed for the first time from different perspectives (Experiments 2, 3).

The anterior thalamic nuclei are assumed to help spatial learning when a particular location is determined by its relationship to multiple, distal cues. This function is revealed when rats with anterior thalamic lesions try to learn specific locations in the Morris watermaze (e.g., Sutherland & Rodriguez, 1989; van Groen et al., 2002; Warburton & Aggleton, 1998; Warburton et al., 1999). Other evidence includes the finding that anterior thalamic nuclei lesions impair item-place associations that involve distal location information, but not item-context associations that involve local information (Dumont et al., 2010, 2014; Gibb et al., 2006; Sziklas & Petrides, 1999). In this respect, the spatial functions of the anterior thalamic nuclei closely parallel those of the hippocampus (Albasser et al., 2013; Dumont et al., 2007; Sziklas et al., 1998), a similarity seen across a range of tasks involving spatial information (Aggleton et al., 1995, 1996; Byatt & Dalrymple-Alford, 1996; Harley, 1979; Law & Smith, 2012; Mitchell & Dalrymple-Alford, 2006; Olton & Papas, 1979; Warburton et al., 1997, 2001).

A widespread assumption is that in many spatial tasks, including those in the Morris watermaze, the rat acquires a map-like representation of the test environment (O’Keefe & Nadel, 1978; Tolman, 1948). Using this cognitive map the rat can flexibly identify the specific arrays of distal stimuli that signal particular locations. There is, however, an inherent problem with many spatial learning tasks as the rat is required to locomote to the goal during training. Consequently, responses made just before reaching the goal are reinforced. Thus, rather than acquiring a cognitive map that reflects stimulus–stimulus learning of the relationships between two or more cues and the goal, animals may simply navigate with reference to a single or limited set of landmarks. In the watermaze, this stimulus–response option is potentially available as swimming in a certain direction relative to a chosen landmark will bring the rat toward its goal (Hamilton et al., 2007; Pearce et al., 1998). The accuracy of the location discrimination is further enhanced if the rat also learns to stop swimming when a particular distance from the landmark (Horne et al., 2012; Pearce et al., 1998). This more inflexible response strategy appears to be shown, for example, by rats with fornix lesions, which nevertheless still display place learning (Eichenbaum et al., 1990).

The present study, which examined the consequences of anterior thalamic lesions, had two principal goals. The first was to encourage a stimulus-stimulus solution in a swimming task. For this reason, rats were trained ‘passively,’ that is, the escape platform location was learnt by placing the rat repeatedly on the platform without being allowed to swim to the escape platform (Horne et al., 2012; Gilroy & Pearce, 2014). Only following training designed to stop stimulus-response learning were rats tested ‘actively,’ that is, allowed to swim to the escape platform. The passive training should also limit procedural learning, which can further impact on swim maze performance (Bannerman et al., 1995; Cain et al., 2006).

The second goal was to compare two different classes of spatial cue. One class concerned the geometrical properties of the environment. For this reason, Experiment 1 used a rectangular pool where different wall lengths created two pairs of corners each with distinctive geometric properties for example, long wall to the left and short wall to the right (see Figure 1). The escape platform was placed in one corner. Previous research has shown that anterior thalamic lesions impair the ability to use geometric cues when trained actively (Aggleton et al., 2009), and so the present experiment did not include the active training condition. The second class of cues (Experiments 2, 3) concerned the visual arrangement of different patterned walls surrounding a square pool. In a square pool, the walls are all the same length and so the identity of a particular corner is determined by the juxtaposition of distinctive walls for example, black wall to the left of white wall (see Figure 1). Again, the escape platform was placed in one corner.[Fig-anchor fig1]

## General Method

The study involved three experiments, each with a different cohort of rats. The first experiment examined passive location learning in a rectangular pool. The next two experiments both compared passive with active learning in a square pool where the appearance of the walls signified the correct location. In all cases, the test pool (rectangular or square) was set within a larger circular pool. The test pool was rotated within the circular pool after every trial to ensure that the rats did not use cues beyond the arena to solve the tasks.

### Subjects

The three experiments involved separate cohorts of adult male Lister Hooded rats (Cohort 1, Harlan, Bicester, U.K.; Cohort 2 and Cohort 3, Charles River, Kent, U.K.). The total numbers of rats were as follows; Experiment 1, *n* = 25; Experiment 2, *n* = 27; Experiment 3, *n* = 25. The rats weighed 270 to 320 g (Exp. 1–3) at the beginning of the experiment and were housed in pairs under a 12-hr light/dark cycle. The animals were given free access to food and water for the duration of the experiments. The rats either sustained bilateral lesions of the anterior thalamic nuclei (ATNx1 = 15; ATNx2 = 14; ATNx3 **=** 10) or sham surgeries (Sham1 = 10; Sham2 = 13; Sham3 = 15). All animals were habituated to handling before the start of the first experiment. All experiments were performed in accordance with the U.K. Animals (Scientific Procedures) Act (1986) and associated guidelines, as well as EU directive 2010/63/EU. The study was also been approved by local ethical review committees at Cardiff University.

### Surgery

For Cohorts 1 and 2 the surgeries were performed under pentobarbitone sodium anesthesia (60 mg/kg i.p., Sigma-Aldrich Company Ltd, Dorset, U.K.). Once anesthetized, the animal was placed in the head-holder of the stereotaxic apparatus (Kopf Instruments, CA) with the incisor bar adjusted to +5.0 relative to the horizontal plane. Following an incision, the scalp was retracted to expose the skull. A craniotomy was made and the dura cut exposing the cortex above the target location. Lesions to the anterior thalamic nuclei were made by injecting 0.12M N-methyl-D-aspartic acid (NMDA; Sigma Chemicals U.K.) dissolved in sterile phosphate buffer (ph 7.4) over two separate sites within one hemisphere with the use of a 1-μl Hamilton syringe (Hamilton, Switzerland) that was attached to a moveable arm mounted on the stereotaxic frame. The lateral and medial sites were infused with 0.22 μl or 0.24 μl of NMDA over a period of five minutes, respectively. The syringe was left in situ for an additional four minutes before being retracted. The lesion coordinates for the ATNx1 group relative to bregma were anteroposterior (AP) −0.6; mediolateral (ML) ± 0.9 and ± 1.8 from the midline; dorso-ventral (DV) −7.0 and −6.3 from bregma for the medial site and the lateral site, respectively (these depth coordinates were changed to −7.1 and −6.4 for the ATNx2 group). For the sham surgeries, the syringe was lowered to + 0.2 above the target site for a few seconds, and then removed. No NMDA was injected in these rats.

Minor refinements were made to the surgical procedures for Cohort 3. For 21 of the 25 rats the surgery was performed under an isoflurane-oxygen mixture (1.5–2.5% isoflurane) with a reduced dose of sodium pentobarbital (14 mg/kg, i.p) when the surgery was nearly completed. For the remaining four rats the surgery was performed entirely under sodium pentobarbital anesthesia (i.e., like Cohorts 1 and 2). The injection site coordinates for Cohort 3 were as follows: medial injections, AP −0.1, ML ± 0.8, DV −6.8; lateral injections, AP −0.4, ML ± 1.5, DV −6.2. Each of the medial injections consisted of 0.20 μl of 0.12M NMDA while the more lateral injections consisted of 0.18 μl of 0.12M NMDA.

After removal of the Hamilton syringe, the incision was cleaned and sutured. A topical antibiotic powder (Aureomycin, Fort Dodge, Animal Health, Southampton, U.K.) was applied. The rats received glucose-saline (5 ml s.c.) for fluid replacement and were then placed in a recovery chamber until they regained consciousness. Rats were given the analgesic Metacam (0.06 ml s.c.; 5 mg/ml meloxicam; Boehringer Ingelheim Vetmedica, Germany). A respiratory stimulant millophylline (0.1 ml s.c., Arnolds Veterinary Products, Shropshire, U.K.), an antimicrobial Baytril in their water (2.5%; Bayer Ltd, Animal Health Division, Ireland), and low dose of diazepam (0.07 ml s.c., 5 mg/ml; CP Pharmaceuticals Ltd, U.K.) was administered to facilitate postoperative recovery as advised. All animals were monitored carefully until they had fully recovered.

### Histology

After behavioral testing, the animals were administered with an intraperitoneal injection of a lethal overdose of Euthatal (200 mg/ml sodium pentobarbital, Marial Animal Health Ltd., Harlow, Essex, U.K.) and perfused intracardially with 0.1M phosphate buffer saline (PBS) followed by 4% paraformaldehyde in 0.1M PBS (PFA). The brains were extracted from the skull and placed on a stirrer to postfix in PFA for four hours, after which the brains were placed in 25% sucrose overnight. The brains were frozen on a microtome (Leica, U.K.) and sectioned at 40 μm in the coronal plane. One-in-five sections were mounted and stained with cresyl violet, a Nissl stain.

### Volumetric Analysis

The extent of the lesions in the anterior thalamic nuclei was first drawn by hand onto five equidistant coronal sections (Paxinos & Watson, 2005). Any unintended hippocampal damage was also plotted onto the appropriate subset of sections from a series of 20 equidistant coronal plates (Paxinos & Watson, 2005). These images were scanned, and the area of damage was quantified using the program analySIS^D (Soft-Imaging Systems, Olympus). The percent damage to the anterior thalamic nuclei and to the hippocampus was quantified by taking the area of damage within the region of interest and dividing it by the total area of that region summed across each drawing.

### Behavioral Testing

Both Cohort 1 and Cohort 2 received other spatial and nonspatial behavioral testing, but none was in a swim pool. Previous tasks given to Cohort 1 involved the following: object recognition memory and object recency memory, T maze alternation, and a contextual biconditional discrimination task (Dumont & Aggleton, 2013; Dumont et al., 2014). Cohort 2 was previously trained on a spatial go/no-go discrimination that involved digging for food rewards in different locations and had also learnt a spatial biconditional problem in a separate room from that used in the current study (Dumont et al., 2014). Cohort 3 had learnt a series of force-choice nonspatial discriminations involving different odors and digging media, along with an automated task based on the Stroop test (Haddon & Killcross, 2005, 2006). The rats were approximately 12 to 13 months (Cohort 1), 10 months (Cohort 2), and 7 to 8 months (Cohort 3) old at the start of the experiments reported below.

## Experiment 1: Passive Place Learning Involving Geometry (Rectangular Pool; Cohort 1)

This experiment determined whether rats with damage to the anterior thalamic nuclei could learn a particular location based on the geometrical properties of the environment. All training was passive as the rats were not allowed to swim to the escape location during acquisition.

### Apparatus and Room

**Figure 1 fig1:**
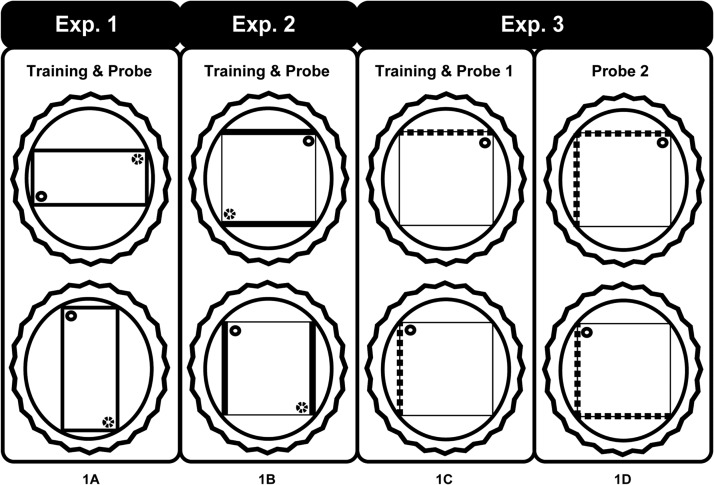
Schematic diagram of the swimming pool tasks run in either a plain rectangle (Experiment 1) or a square with different patterned walls (Experiments 2, 3). The inner shape depicts the pool, the surrounding circle is the larger pool within which the smaller pool is placed, and the rippled circle represents the curtains used to block distal cues. Each inner pool was rotated on consecutive trials, as indicated in the figure. For the square pool the thick dark lines represent black walls (Experiment 2), whereas the broken lines represent striped walls (Experiment 3). In Experiment 3 the rats received an additional probe in which the wall configuration was changed from one striped wall to two, adjacent striped walls (1D). The small circle represents the platform where a rat would be placed passively. The small dotted circle represents the other identical and, hence, other correct corner.

A white, circular swim pool, measuring 200 cm in diameter and 60 cm deep, was fixed 60 cm above the floor in the center of a room (400 cm × 400 cm × 230 cm; Room A). The pool was filled with water to a depth of 27 cm and was maintained at a temperature of 25 °C (± 2 °C). The water was made opaque by adding 0.5 L of white opacifier (Opulyn 303B, Dow; Cat No. 10318500), which was changed daily. Throughout the experiment, rats were trained in a rectangular-shaped pool set within the circular pool (Figure 1A). This rectangular pool was constructed from two gray, long Perspex boards (180 cm long, 59 cm high, and 2 mm thick) and two gray, short Perspex boards (90 cm long, 59 cm high, and 2 mm thick). Each board was placed vertically in the pool and suspended by bars that extended over the edge of the pool.

A white circular false-ceiling (200 cm in diameter) was suspended 175 cm above the floor of the pool. A video camera fixed to the center of the ceiling recorded the rats’ movements, which were analyzed using Watermaze software (Morris & Spooner, 1990). Eight, 45-W lights (22.5 cm in diameter) located in the circular ceiling illuminated the pool. The lights were equidistant from each other in a 160-cm diameter circle, whose center was the same as the center of the circular ceiling. The training room was also lit by two 153-cm strip lights connected end to end on each of the East and West walls. These lights ran parallel with the floor and were situated 75 cm above the floor. An escape platform (10 cm in diameter) was mounted on a column that rested on the bottom of the pool, which resulted in the top of the escape platform being submerged 2 cm below the surface of the water. A white curtain, which was attached to the edge of the circular ceiling, was drawn completely around the pool during all training and test trials, so hiding distal room cues, including the strip lights. The curtain was 150 cm high and fell 25 cm below the edge of the pool. There was a door (175 cm by 200 cm) in the center of the South wall connecting the room with the swim pool to the room containing the computer equipment used to monitor the rats’ behavior.

### Procedure

The ATNx1 and Sham1 rats completed one session of four training trials each day. For each session they were carried into a room adjacent to the test room in groups of five in a light-tight aluminum carrying box and remained in this box between trials. For each trial, the rat was carried from the box to the pool and placed on the platform. The rat was allowed to stay on the platform for 30 s, undisturbed, before being removed, dried and returned to the holding box.

### Pretraining

Pretraining (three sessions) was designed to discourage the rats from stepping off the platform during the placement sessions. For these sessions the escape platform was placed in a quadrant (NE, NW, SW, or SE) in the circular pool, that is, not the rectangular pool used in the experiment proper. Each location was used once in a session. The platform was randomly positioned either 25 cm or 50 cm from the edge of the pool, each for two trials per session. The rats were placed on the platform for 30 s. If a rat stepped off the platform and did not immediately climb back unto the platform, the experimenter indicated the location of the platform by tapping on the escape platform. If the rat still failed to return to the platform, the experimenter would guide the rat (they would follow the experimenter’s hand through the water) back to the platform, where the rat remained for 30 s.

### Training

The rats next received 12 sessions of training in the rectangular pool (Figure 1A). The platform was positioned 25 cm from a corner on an imaginary line that bisected the corner. The position of the platform was counterbalanced, so that half of the rats from each group had the platform placed in a corner where the short wall was to the right of the long walls and the other half received the platform in the corner where the short wall was to the left of the long wall (see Figure 1A). Between each trial, the rectangular pool was randomly rotated 90°, 180°, or 270° clockwise. Four possible orientations were used (North, South, East, or West) with each orientation being used once for any given session (see Figure 1A). Similar to pretraining, the rats were placed on an escape platform. If any rat fell into the pool and failed to climb back onto the platform immediately, the experimenter would remove the rat from the pool and return it to the platform.

The first three trials of the final session, Session 12, were conducted in the same manner as previous trials. The fourth trial consisted of a Probe Test. The platform was removed and the rats were released into the water in the center of the rectangle, facing away from the experimenter. Each rat was allowed to swim for 60 s.

### Statistical Analysis

The first corner each rat approached in the Probe Test was recorded (i.e., correct or incorrect corner) as well as the time taken to first swim to one of the two possible correct corners. Circular search zones in each of the four corners were then used to analyze further the results from the test trial. Each zone had a diameter of 30 cm with its center positioned 25 cm from a corner on a line that bisected the corner. The percentage of time spent in the correct zones (i.e., the corner where the platform was located during training, and its geometrically equivalent and diametrically opposite corner—hence, also correct) and incorrect zones (the remaining two corners) of the rectangular pool were analyzed using one between-subjects factor (Group) by one within-subject factor (Corner: correct; incorrect) ANOVA. The mean swim speed (cm/s) and the mean distance traveled (cm) were also examined using *t* tests to compare the ATNx1 and Sham1 groups (two-tailed). The nonparametric Mann–Whitney test was used when the data violated the assumptions of parametric tests (e.g., normality). Nonparametric tests were also used to analyze the first corner choice data. Binomial tests helped to determine whether each group was significantly above chance in their first choice of escape corner (all one-tailed analyses), whereas Fisher’s Exact Probability was used to compare whether the two groups differed significantly from one another (no directional hypotheses, therefore, two-tailed analyses).

## Experiment 2A: Passive Place Learning With Black-White Walls (Square Pool; Cohort 2)

This experiment examined whether rats with lesions in the anterior thalamic nuclei could passively learn a location that depended on the different arrangement of black-white walls.

### Apparatus and Room

The rats were trained in a square pool in a room different from Experiment 1 (430 cm × 400 cm × 240 cm; Room B). Throughout the experiment proper, the square pool was constructed from two white Perspex boards (140 cm long, 50 cm high, and 2 mm thick) and two black Perspex boards (140 cm long, 53 cm high, and 2 mm thick). Each board was placed vertically in the circular pool and suspended by bars that extended over the edge of the pool, and alternated between black and white walls. This configuration created two pairs of different corners: a) where the black wall was to the left of the white wall, and b) where the white wall was to the left of the black wall (Figure 1B). All other aspects of the apparatus were identical to those described for Experiment 1.

The pretraining procedure was different from Experiment 1 (details below) as during the first two sessions of pretraining, a beacon (i.e., a landmark) was attached to the platform. The beacon was a stick (15 cm high and a 4 cm diameter) with alternating black and white stripes (2 cm). As in Experiment 1, a white curtain was drawn completely around the pool during all training and test trials, so hiding distal cues.

### Pretraining

The change in pretraining from Experiment 1 was to provide active training, as there was a subsequent, active training component in both Experiments 2 and 3. This pretraining protocol also made it possible to acquire additional information concerning baseline measures of swimming, orientating, and escape motivation. The escape platform was placed within the circular pool and moved in a manner identical to Experiment 1. However, there were two differences in the pretraining: a) In Experiment 2, the first two pretraining sessions included a beacon to help locate the platform, and b) the rats were required to actively swim and climb onto the escape platform (in Experiment 1, the rats were placed passively onto the platform). As a result, the rats were released from one of eight start positions (W, E, S, N, NE, NW, SW, or SE), with each location used twice throughout the four sessions, but not within the same session. The rats had a maximum of 120 s to find the platform on the first three sessions, and 90 s during Session 4. If the rats successfully found the platform, they remained on the platform for 30 s before being returned to the carrying box. However, if the rats did not find the platform, the experimenter showed the rat the location of the platform as described in Experiment 1. The rats completed one session of four training trials each day.

### Training

The rats received 12 sessions, each with four training trials, in the square pool. The platform was positioned 25 cm from a corner on an imaginary line that bisected the corner (Figure 1B). The position of the platform was counterbalanced so that half of the rats from each group had the platform placed in a corner where the adjacent black wall was to the left of the adjacent white wall. The remaining rats received the opposite wall arrangement. Between each trial, the square was randomly rotated 90°, 180°, or 270° clockwise. As in Experiment 1, the rats were placed on the platform for 30s, facing the corner, before being returned to the metal carrying box.

The first three trials of the final session, Session 12, were conducted in the same manner as previous trials. The fourth trial consisted of a Probe Test in which rats were released into the center of the square pool and allowed to swim for 60s.

## Experiment 2B: Active Place Learning With Black-White Walls (Square Pool; Cohort 2)

This experiment determined whether rats with anterior thalamic nuclei lesions could solve the black-white place learning task (Experiment 2A) when allowed to navigate actively to the goal location during training. Unlike Experiment 2A, two identical escape platforms were used because there were two correct corners (Figure 1B)

### Procedure

Each rat was placed in the center of the pool with the experimenter initially stood in one of four locations (N, E, S, W) in a session. The rat was given 60 s to swim to one of the two platforms located in the correct corners (i.e., the same black-white configuration that they had previously experience during Experiment 2A). Each of the four start locations was used once per session. At the end of 60 s, if the rat had not located the platform, the experimenter indicated the location of the platform to the rat in the same way as during pretraining (Experiment 2A). The rat remained on the platform for 30 s before being returned to the metal carrying box. The rats received a total of seven sessions. On the final trial of Session 7, the rats were given a Probe Test. The rats were placed in the center of the pool and allowed to swim for 60 s in the square pool without any escape platforms present.

## Experiment 2C: T Maze Alternation (Cohort 2)

To test the effectiveness of the surgeries in Cohort 2, these rats were tested on a spatial working memory task known to be sensitive to anterior thalamic damage (e.g., Aggleton et al., 1995, 1996; Loukavenko et al., 2007).

### Apparatus and Room

Pretraining and testing took place in a different room (304 cm × 290 cm × 239 cm; Room C). The room contained a variety of extramaze cues (e.g., posters, tables, door) and was illuminated by two fluorescent strip lights (140.8 lux in the center of the room). Two identical cross-mazes were used. The walls of each of the four arms of the two mazes (45.5 cm long × 12.0 cm wide × 32.5 cm high) were made of black Perspex. The floor of the two mazes were made of wood and painted white. A sunken food-well (2 cm in diameter and 0.75 cm deep) was located at the end of each arm. By placing an aluminum barrier at the entrance of an arm it was possible to prevent access to that arm. The mazes were placed on a table 74 cm high. During pretraining the mazes were placed side by side so that the East arm of the left maze (Maze A) touched the West arm of the right maze (Maze B). However, during the test proper, only one maze was used per session and so it was placed in the center of the table. Each of the two mazes was used on alternate days.

### Pretraining

During both pretraining and testing, the rats were transported inside a light-tight aluminum carrying box where they also remained between trials. During pretraining, the apparatus was blocked at the central junction with a metal barrier, creating three straight alleys: a) a start arm alley (South arm), b) both the choice arms (i.e., the top of the “T”; East and West arms), and c) the North arm (opposite the start). Rats were trained to eat in these straight alleys, and so not rewarded for specific arm turns. Each rat was placed for five minutes in a potential alley with sucrose pellets (45 mg per pellet; Noyes Purified Rodent Diet, Lancaster, NH), initially scattered along the floor, but later placed within the food-wells. Over the four days, every rat experienced the two mazes and the various maze arms.

### Testing

The rats received six trials per day for eight days. Each trial consisted of both a sample phase and a choice phase. During the sample phase, the rat was allowed to enter just one of the arms at the top of the “T” by blocking the entrance to the opposite arm at the central junction in the maze. The rat was then allowed to consume the single sucrose pellet at the end of the sample arm. The rat was then picked up and confined in the start arm for approximately 15 s while the barrier at the choice point was removed. The metal barrier at the start area was then removed to begin the choice phase, where the rat had free access to the two arms of the T maze. The rat was rewarded with a single sucrose pellet for choosing the arm that was not previously visited during the sample phase (i.e., the rat alternated arms between the sample and choice runs). The rat was deemed to have made a choice when it placed a hind foot down an arm. After a correct choice, the rat was allowed to eat the reward before being returned to the metal carrying case. When the rat made an incorrect choice, it was allowed to run down the entire length of the arm to reach the empty food-well before being returned to the carrying case. The rats were run in squads of 3 to 4, each rat receiving one trial at a time. Consequently, the intertrial interval was approximately 4 to 5 minutes.

## Experiment 3A: Passive Place Learning With Striped - White Walls (Square Pool; Cohort 3)

This experiment examined whether rats with lesions to the anterior thalamic nuclei could passively learn a location that depended on the different arrangement of black and white striped walls compared with white walls, for example, to distinguish the corner with a white wall to the left and a striped black/white wall to the right (Figure 1C). A single striped wall replaced the two black walls used in Experiment 2 (see Figure 1). The change in wall color reflected the finding that most rats appeared to have a spontaneous preference for darker walls. The change to a single contrasting (striped) wall meant that only one of the four corners was correct (rather than two of the four corners in Experiments 1 and 2).

### Apparatus and Room

The rats were pretrained in a circular pool in Room D (365 cm × 305 cm × 240 cm), and all subsequent testing was conducted in Room B (430 cm × 400 cm × 240 cm) that is, the same pool and room as used in Experiment 2. Throughout the experiment, rats were trained in a square-shaped pool constructed of three white Perspex boards (140 cm long, 50 cm high, and 2 mm thick) and one black and white striped Perspex board (140 cm long, 53 cm high, and 2 mm thick). The vertical black stripes were 10 cm wide with 10-cm white intervals between stripes. The black stripes began 5 cm from the side edge of the board. The test configuration (Figure 1C) created three sets of corners: a) black and white striped wall to the left of the white wall, b) black and white striped wall to the right of the white wall, and c) white wall meeting white wall (two of these corners; see Figure 1C).

### Procedure

#### Training and first probe – one striped wall

After pretraining (see Experiment 2A), the rats were passively trained for eight days, each with four training trials, in the square pool. The procedure matched that described for Experiment 2A. On the eighth day (final session), the rats received three training trials followed by the first Probe Test, where the platform was removed and the animal was allowed to swim for 60 s. After the first Probe Test, the animal received one more passive training day (four trials) before a further session with a Probe Test on the fourth trial (i.e., ‘Retest’). In all other respects training matched that for Experiment 2A.

#### Training and transfer probe - two striped walls

The rats were passively trained for an additional two days as described above with one striped wall, before on the third day receiving a probe (Test trial) that now used two black and white striped walls arranged next to each other for the first time (Figure 1D). The first three trials involved standard passive training with the one striped wall, but this was replaced by the two striped condition on the fourth trial (Figure 1D) when the rat was put into the water in the center of the pool. This new configuration created four different corners: a) black and white striped wall to the left of the white wall, b) black and white striped wall to the right of the white wall, c) junction of two striped walls, and d) junction of two white walls. In all other respects, the probe tests were identical to those in the one striped wall condition. The animals were again given an additional day of passive training (one striped wall) before repeating the two striped wall probe test on the fourth trial of the next session (i.e., Retest).

## Experiment 3B: Active Place Learning With Striped - White Walls (Square Pool; Cohort 3)

This experiment examined whether rats with lesions to the anterior thalamic nuclei could find the correct corner when allowed to swim to the goal location during training, that is, active training. The room and apparatus were the same as Experiment 3A.

### Procedure

The procedure was similar to Experiment 3A, with the exception that the rat was released from one location (South) into the center of the pool and given 60 s to swim to a platform located in the correct corner (i.e., the same black and white striped wall compared with white wall configuration that they had previously experienced during Experiment 3A). The rats received four days of active training, with four trials per day. After each trial the walls were rotated 90^o^ anticlockwise so the direction to the platform from the release point was always different. Having climbed onto the platform, the rat remained there for 30 s before being returned to the metal carrying box. On the final trial of the fourth day, the rats were given a single Probe Test. Each rat was placed in the center of the pool and allowed to swim for 60 s without any platform present.

## Results

### Histology

**Figure 2 fig2:**
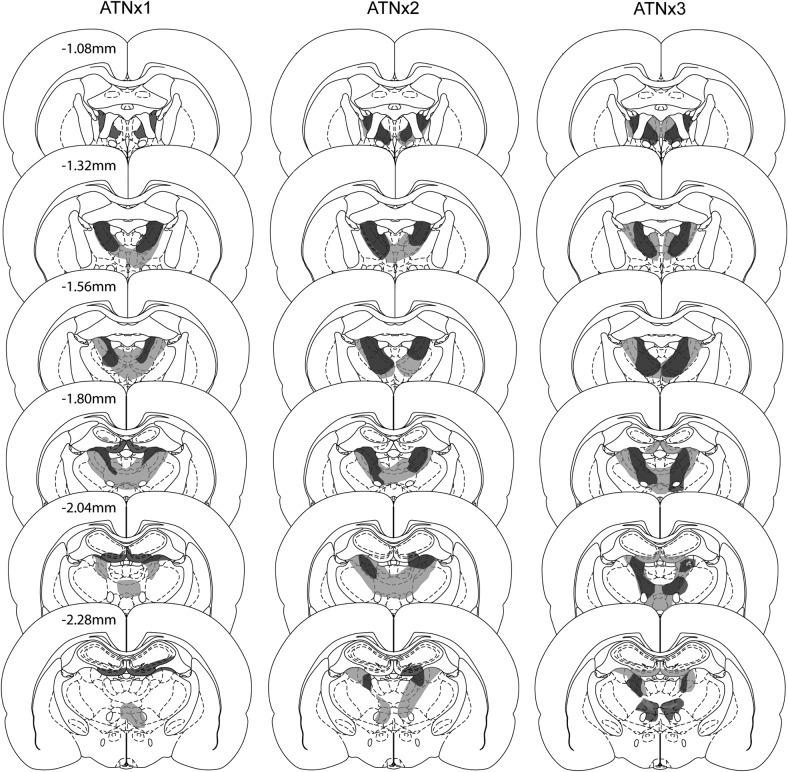
The figure depicts the minimum (dark gray) and maximum (light gray) extent of the lesions for the ATNx1, ATNx2, and ANTx3 groups on a series of coronal sections. The numbers refer to the approximate distance in mm of each section caudal to bregma. Sections adapted from *The Rat Brain in Stereotaxic Coordinates* (5th ed.), Figures 42, 44, 46, 48, 50, and 52, by G. Paxinos and C. Watson, 2005, New York, NY: Academic Press. Copyright 2005 by Elsevier Academic Press. Adapted with permission.

The smallest and largest anterior thalamic lesions in the three cohorts are shown in Figure 2.[Fig-anchor fig2]

#### Cohort 1

Three ATNx1 animals were excluded as more than 50% of the ATN was spared. For the remaining 12 ATNx1 rats the total area of cell loss in the anterior thalamic nuclei was between 52% - 94% (*M* = 76%; median = 76%). Any sparing typically occurred within the caudal anterior thalamic nuclei, often in the most ventral portion of the anterior medial nucleus. However, two rats exhibited the opposite pattern with a more complete lesion at the caudal end of the anterior thalamic nuclei, with sparing occurring rostrally. These two animals had some sparing to the anterior dorsal nucleus. In 11 of 12 cases, there was partial damage to the rostral and dorsal portions of the laterodorsal nucleus, which in three cases was unilateral. In those rats with larger lesions, there was also some restricted damage to the parataenial nucleus (*n* = 7; unilateral in two cases), the paraventricular nucleus of the thalamus (*n* = 3), the reticular nucleus (*n* = 6; unilateral in three cases), and nucleus reuniens (*n* = 7).

In all cases there was some isolated cell loss in the hippocampus. This hippocampal damage was largely restricted to the very rostral (septal) part of the ventral (inferior) blade of the dentate gyrus. Of the 12 cases, 11 had restricted bilateral damage to just this part of the septal dentate gyrus, whereas in one case it was unilateral. In some cases this cell loss extended into the immediately adjacent CA3 (*n* = 9; of which three had unilateral cell loss). It is important to stress that this hippocampal damage was very limited, producing a mean loss of 3.3% of the total hippocampus (range, 0.2% to 5.8%). In one case the injection tracts in the fornix appeared to induce some additional damage (primarily unilateral), whereas in three other cases the fornix appeared intact but slightly distorted in both hemispheres.

#### Cohort 2

Four ATNx2 rats were excluded from further analysis. In three of these cases, there was excessive sparing of the anterior thalamus and in one further case the lesion extended into the medial septal nuclei. In the remaining 10 cases (see Figure 2) there was considerable cell loss in the anterior thalamic nuclei with the lesion occupying from 73% to 100% (*M* = 93%; median = 96%) of the area. In those cases with smaller lesions, sparing typically occurred in the anterior medial nucleus in one hemisphere. In all 10 cases, the posterior extent of the lesion encroached into the most rostral and dorsal portions of the laterodorsal nucleus and in five cases the lesions reached the rostral cap of the medial dorsal nucleus of the thalamus (only unilateral in three cases). In some cases there was partial damage to the parataenial nucleus (*n* = 8), the paraventricular nucleus (*n* = 5), the reticular nucleus (*n* = 8, unilateral in two cases), nucleus reuniens (*n* = 9), and the ventral anterior thalamic nucleus (*n* = 9, unilateral in four cases).

In five cases there was some restricted bilateral cell loss in the hippocampus; three other rats had restricted unilateral damage to this region. The cell loss was typically confined to the most rostral part of the ventral (inferior) blade of the dentate gyrus (*n* = 8, in three cases the damage was unilateral), but occasionally the atrophy extended into the immediately adjacent part of CA3 (unilateral *n* = 2, bilateral *n* = 1). In three cases, the damage reached the medial part of septal CA1 (unilateral *n* = 2, bilateral *n* = 1). A mean of 1.5% of the total hippocampus was damaged (range 0% - 5.8%). In one case there was unilateral distortion of the fornix.

#### Cohort 3

Of the 10 rats, two had excessive, unintended cell loss within the medial dentate gyrus of the septal hippocampus, and were excluded. All subsequent descriptions relate to the remaining eight ATNx3 rats (see Figure 2). In these eight cases, the anterior thalamic nuclei lesions were either essentially complete (*n* = 4) or a small island of cells within the anterior ventral nucleus was visible in just one hemisphere. Consequently the range of tissue loss from the anterior thalamic nuclei was 82% - 98% (*M* = 92%; median = 95%). The lesions typically extended into adjacent midline nuclei such as the paraventricular nucleus (*n* = 4) and parataenial nucleus (*n* = 4, three of which had only unilateral cell loss). The lesions also extending ventrally to reach the very rostral part of the reticular nucleus and the ventral anterior nucleus (both, *n* = 3). The rostral nucleus reuniens was involved seven cases. More caudal nuclei such as the medial dorsal thalamic nucleus (unilateral, three cases) and the lateral dorsal nucleus (three cases, two of which unilateral) were occasionally involved at their rostral limit. Cell loss within the hippocampus was seen in only three cases, where it was typically restricted to the ventral blade of the dentate gyrus in the most rostral part of the septal hippocampus. Consequently, hippocampal damage ranged from 0% to 3.3% (*M* = 0.8%; median = 0%). A more common feature was that the third and lateral ventricles appeared enlarged.

### Behavioral Findings: Experiment 1

Three Sham1 rats stepped off the platform once during a training trial and so experienced a limited swim (e.g., circling the platform in an attempt to climb back on it). Inspection of the subsequent probe data did not indicate that this experience influenced performance, so these three Sham1 rats were included in the analyses.

**Figure 3 fig3:**
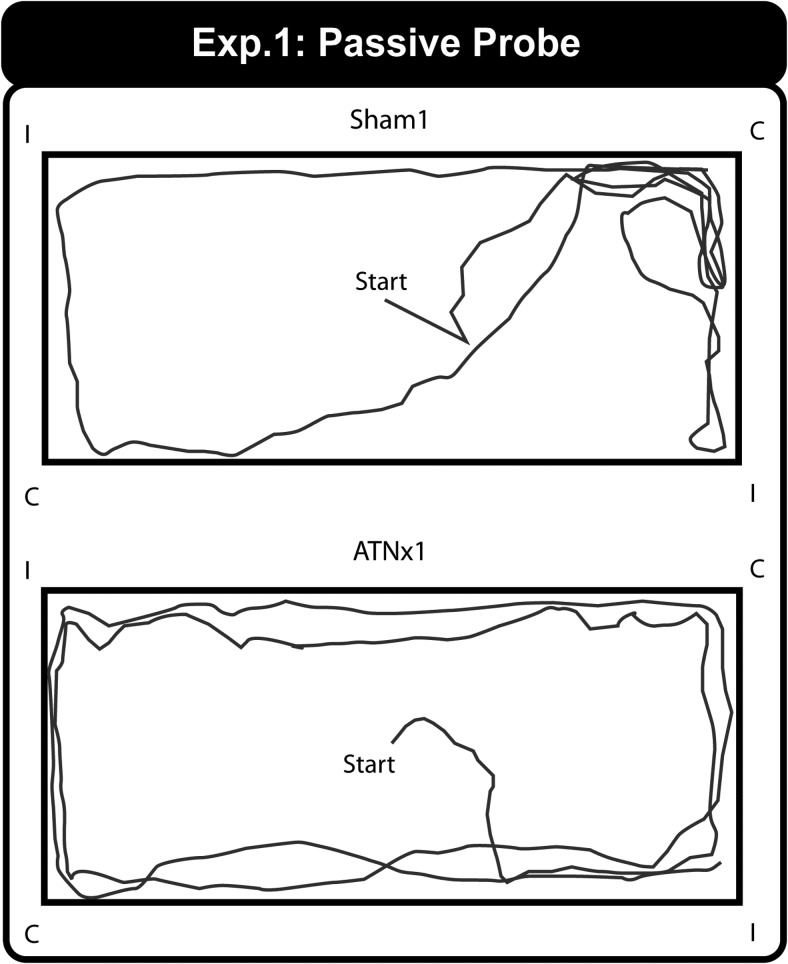
Experiment 1 – Rectangular Pool. Representative swim paths during the 60-s probe for a Sham1 (upper) and ATNx1 (lower) animal. For the Probe Test the escape platform was removed. C = correct corner; I = incorrect corner.

On the single probe the rats were placed in the center of the rectangular pool and allowed to swim for 60 s. Examples of the swim paths for a Sham1 and ATNx1 rat during the one minute test trial (i.e., without the presence of the platform) are shown in Figure 3.[Fig-anchor fig3]

Eighty percent of the Sham1 rats swam to the correct corner first, whereas only 50% of the ATNx1 group went to the correct corner first. Binomial tests indicated that the Sham1 group swam to the correct corner first, although this choice measure narrowly failed to reach significance (*p* = .055; one-tailed). The TNx1 group showed no evidence of a correct preference (*p* > .1; one-tailed). Although the groups did not differ significantly from one another on this choice measure (Fisher’s Exact Probability, *p* > .1), the Sham1 group took significantly less time to swim to the correct corner (*M* = 6.8s, SEM = 1.9s) than the ATNx1 rats (*M* = 13.1 s, *SEM* = 2.2s) (Mann–Whitney U Statistic = 24.5, *p* = .02). A nonparametric test was used because of a violation of normality in these latency data.

**Figure 4 fig4:**
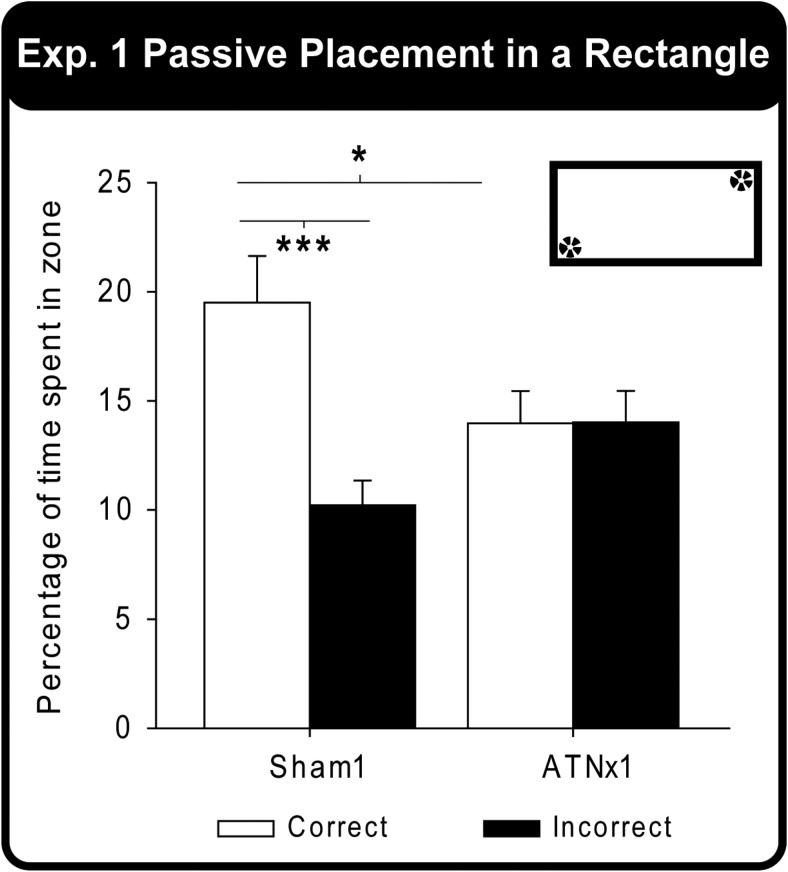
Experiment 1 – Rectangular Pool. Percentage of time spent swimming in either the correct or incorrect zones (corners) for both the Sham1 and ATNx1 groups during the Probe Test. Data shown are group means, and the vertical bars are the standard error of the means (*SEM*). * *p* < .05, ** *p* < .01. The inset shows that there are two ‘correct’ locations but that the platforms are absent.

Figure 4 displays the percent time the Sham1 and ATNx1 rats spent in the two correct corners (the two diametrically opposite corner where the rats had been placed during acquisition) and the two incorrect corners during the single Probe Test. A two-way mixed model ANOVA yielded a significant Group × Corner interaction, *F*(1, 20) = 8.21, *p* = .01. Examination of the simple effects indicated that the Sham1 group spent significantly more time in the correct corners compared with the incorrect corners, *F*(1, 20) = 16.3, *p* = .001, whereas the ATNx1 group did not (*p* > .1). Although the Sham1 group spent significantly more total time in the correct corners compared with the ATNx1 animals, *F*(1, 40) = 6.14, *p* = .018), the two groups did not differ significantly in the time spent in the incorrect corners, *F*(1, 40) = 2.89, *p* = .098; Figure 4). Finally, there were no group differences in the mean distance traveled (cm) or the mean swim speed (cm/s) of the animals during the Probe Test (both *p* > .1).[Fig-anchor fig4]

### Behavioral Findings: Experiment 2A

#### Pretraining

The mean latency to find the platform, the mean distance traveled, and the mean swim speed for the ATNx2 and the Sham2 rats did not yield any significant group differences during pretraining (all, *p* > .1). None of the interactions involving the lesion group were significant (*p* > .05), and although there was a suggestion of a Group × Condition (*p* = .075) and a Group × Day (*p* = .085) interaction for mean swim speeds, the swim speed scores of the two groups were almost identical for the last two days of pretraining.

#### Probe test

Throughout passive training all of the rats stayed on the platform. For the first active trial, the rats were placed in the center of the square pool and allowed to swim for 60 s with the platform removed. Twelve of the 13 (92%) Sham2 rats swam to the correct corner first, whereas only five of the 10 (50%) ATNx2 rats did. Binomial tests (chance 50%) revealed that the Sham2 group swam to the correct zone significantly more often than predicted by chance (*p* = .0015; one-tailed), whereas the ATNx2 group did not. There was a marginal difference between the two groups (Fisher’s Exact Probability, *p* = .052; two-tailed). The ATNx2 group took significantly longer to swim to the correct corner compared with the Sham2 group, *t*(21) = 2.82, *p* = .01 [means for Sham2 = 12.7 s (*SEM* = 2.3 s), ATNx2 = 26.2 s (*SEM* = 4.6 s)].

**Figure 5 fig5:**
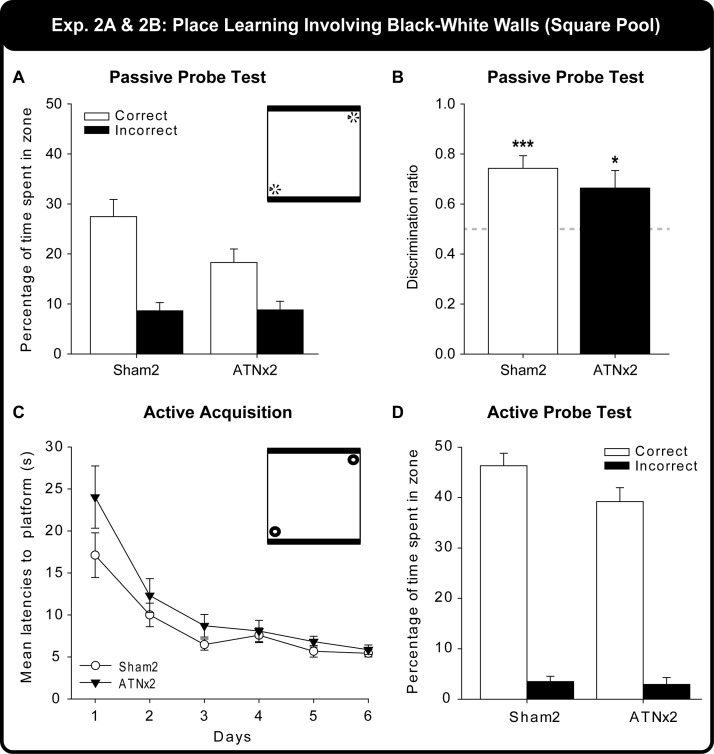
Experiment 2 – Square Pool. The upper graphs (A and B) show performance on the first Probe Test (60 s) after passive training in the pool with two black walls and two white walls. A) Percent of the 60s swim probe spent in the correct and incorrect locations. B) Ratio of time spent in correct over incorrect corner. The lower graphs (C and D) show performance during and after active training, that is, being allowed to swim to the escape location. C) Acquisition performance measured by latency to escape over successive days of training. D) Performance on Probe Test (60 s) after active training showing clear corner preferences. The vignettes show that for A (and for B and D) the platforms are removed, whereas for C the platforms are present. Data shown are group means, whereas the vertical bars are the standard error of the means (*SEM*).

Figure 5A displays the percent time the Sham2 and ATNx2 rats spent in the two correct corners and the two incorrect corners during the Probe Test. The ATNx2 and the Sham2 rats spent a similar amount of time in the incorrect corners (Figure 5A), but the Sham2 rats spent more time in the correct corners compared with the ATNx2 group. A two-way mixed model ANOVA (Group × Corner) yielded a significant main effect of Group, *F*(1, 21) = 5.28, *p* = .032, indicating that the Sham2 group accumulated significantly more time swimming in the four corners than the ATNx2 group. There was also a significant main effect of Corner, *F*(1, 21) = 21.0, *p* < .001). The Group × Corner interaction was, however, not significant (*p* > .1). Finally, the groups did not differ significantly on either their mean swim speeds or on the mean distance traveled during the Probe Test (both, *p* > .1).[Fig-anchor fig5]

Because the total time spent in the four corners differed between the Sham2 and the ATNx2 groups, the data were reexamined using a discrimination ratio (total time spent in the correct corners divided by the total time spent in all four corners, so producing a score between 0 and 1, where chance is 0.5). The discrimination ratios of the ATNx2 and the Sham2 groups (Figure 5B) did not differ significantly (between-sample *t* test, *p* > .1), with both groups spending more time in the correct corner that is, a ratio above 0.5 (one-sample *t* tests Sham2: *t*(12) = 4.81, *p* < .001; ATNx2: *t*(9) = 2.32, *p* = .045).

### Behavioral Findings: Experiment 2B

#### Acquisition

Figure 5C shows the mean latencies to the escape platform by the Sham2 and the ATNx2 groups during the six acquisition days where the rats were allowed to swim to the platform. Both the Sham2 and ATNx2 rats significantly improved as indicated by a decrease in the mean latencies over days, *F*(5, 105) = 26.3, *p* < .001. There was also a borderline effect of group, *F*(1, 21) = 4.21, *p* = .053. Inspection of the figure indicates that the ATNx2 group were slower compared to the Sham2 group on Day 1 (presumably reflecting their greater positive transfer), and this difference was supported by the simple effects, *F*(1, 126) = 9.26, *p* = .003. This group difference disappeared over the following days. The Group × Days interaction was not significant (*p* > .1).

#### Probe test (active)

On the final trial the rats were released in the center of the square pool. As 100% of the rats swam first to the correct zone, both groups were above chance (both *p* ≤ .001, binomial) and did not differ. The Sham2 rats took a mean of 3.7s (SEM = 0.3s) and the ATNx2 group took a mean 4.3s (SEM = 0.5s) to swim to the correct corner, and again the groups did not differ (*p* > .1). The percentage of swim time spent in the correct and incorrect zones of the Sham2 and ATNx2 groups are shown in Figure 5D. A mixed model ANOVA (Group × Zone) yielded a borderline main effect of Group, *F*(1, 21) = 4.25, *p* = .052, a significant main effect of Corner, *F*(1, 21) = 321.8, *p* < .001, but no Group × Corner interaction (*p* > .1). Finally, there was no apparent difference between the Sham2 and ATNx2 mean swim speeds or mean distance traveled (both *p* > .1).

### Behavioral Findings: Experiment 2C

**Figure 6 fig6:**
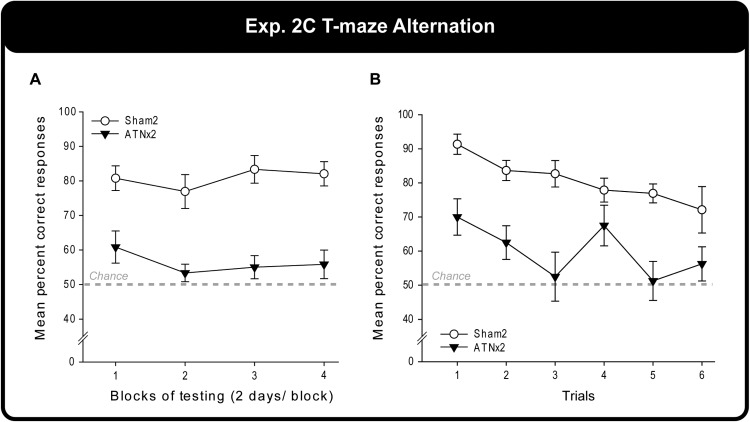
Experiment 2c – Alternation in a T maze. Graph A shows the percent correct response over successive pairs of training sessions by rats the anterior thalamic lesions (ATNx2) and their controls (Sham2). Graph B shows performance as measured by the position of the trial within each daily sequence (1 = the first trial of each day). Data shown are group means, and the vertical bars are the standard error of the means (*SEM*).

The Sham2 rats outperformed the ATNx2 group across testing. A two-way mixed model ANOVA confirmed the deficit in the ATNx2 rats (main effect of Group, *F*(1, 21) = 52.6, *p* < .001), but there was no effect of training (Blocks, *p* > .1)]. Simple effects confirmed that the ATNx2 rats were significantly impaired on every block of testing (all *p* ≤ .001; Figure 6A).[Fig-anchor fig6]

The correct responses of the ATNx2 and Sham2 rats were also examined on a trial by trial basis (Figure 6B) with the data from the various sessions combined for each trial. In addition to the main effect of Group, *F*(1, 21) = 27.8, *p* < .001, there was a main effect of Trial, *F*(5, 105) = 4.37, *p* = .001. The Trial effect reflected the fall in performance from Trials 1 to 6, which matched the rise in proactive interference. The Group × Trial interaction was not significant (*p* > .1), although this analysis was potentially affected by floor effects in the ATNx2 group.

### Behavioral Findings: Experiment 3A

#### Pretraining

Neither the latencies to the submerged platform or the mean swim speeds of the ATNx3 and Sham3 rats differed significantly (latencies, *F*(1, 21) = 3.57, *p* = .073; swim speed, *p* > .1). However, the ATNx3 group did travel further than the Sham3 rats, *F*(1, 21) = 6.87, *p* = .016, though no individual day was significantly different between the two groups. None of the interactions involving the factor Group, that is, with Day or Condition (beacon or no beacon) was significant (lowest *p* = .08).

#### Probe test (passive) – one striped wall, three white walls

**Figure 7 fig7:**
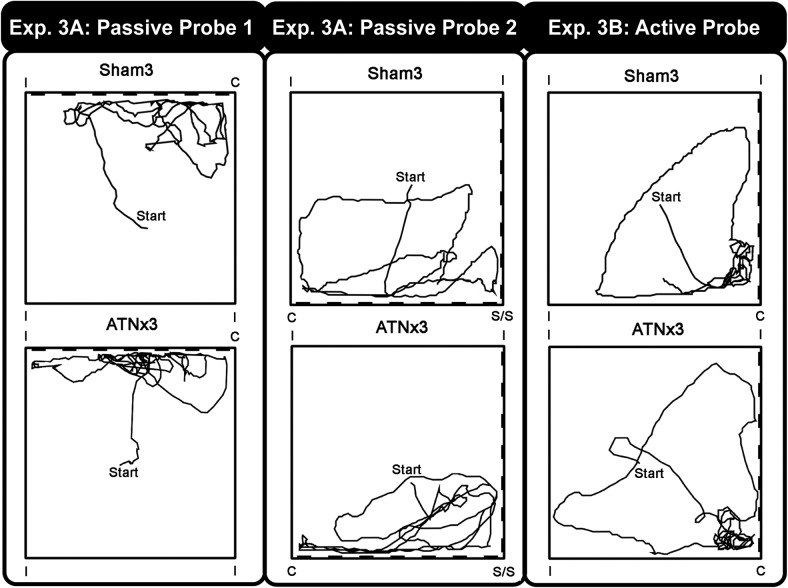
Experiment 3 – Square Pool. The figure shows representative swim paths for a sham control (upper) and a rat with anterior thalamic lesions (lower). The Passive Probe 1 is the first swim in the pool after passive training with one striped wall and three white walls (The broken thick line represents the striped wall). The Passive Probe 2 shows the animal’s first swim behavior when two adjacent patterned walls are introduced. The far right shows probe trial swim paths after active training (‘Active Probe’). For the probe trials the escape platform was removed. Note that the pool is rotated between trials so that the correct corner moves with respect to the outside room. C = correct corner; I = incorrect corner.

The rats were tested twice on this probe (see Methods above). Figure 7 (left panel) shows the swim paths for a Sham3 and an ATNx3 rat from the first of the 60-s probes. For this probe, only one of the four corners is correct (Figure 1C).[Fig-anchor fig7]

On the initial Probe Test, 50% of the ATNx3 and 73% of the Sham3 rats swam first to the correct corner. It is notable that none of the rats first selected a white—white corner. Consequently, the ATNx3 rats equally selected the correct striped-white corner and its mirror image, that is, 50% of ATNx3 rats first swam to the wrong white-striped corner, as compared with 27% of the Sham3 rats. During Retest, 75% of the ATNx3 rats now approached the correct corner first, whereas the performance of the Sham3 group remained the same (73%). With 11 of the 15 Sham3 rats first selecting the correct corner, their choice performance approached significance (*p* = .059) if the binomial test assumes a chance level of 50%, that is, the two white-white corners are discounted.

The mean times taken to swim to the correct corner during the Probe Test and Retest, respectively, were for the Sham3 Group 11.9 s (*SEM* = 1.9s) and 9.5 s (*SEM* = 1.4 s) and for the ATNx3 Group 22.9 s (*SEM* = 7.2 s) and 10.5 s (*SEM* = 2.2s). A mixed ANOVA yielded a significant main effect of Probe, *F*(1, 21) = 5.88, *p* = .025, as the latencies decreased between the Test and Retest. The main effect of Group failed, however, to reach significance, *F*(1, 21) = 3.36, *p* = .081. Because the first probe is unique (it is the only trial to occur before any active training) it is informative to compare the ‘escape’ latencies from just this probe. On this measure, the ATNx3 rats required more time to find the correct zone compared with the Sham3 group, *F*(1, 42) = 6.04, *p* = .018, simple effects. There was, however, no difference on the Probe Retest (*p* > .1), nor was the Group × Probe interaction significant (*p* > .1).

**Figure 8 fig8:**
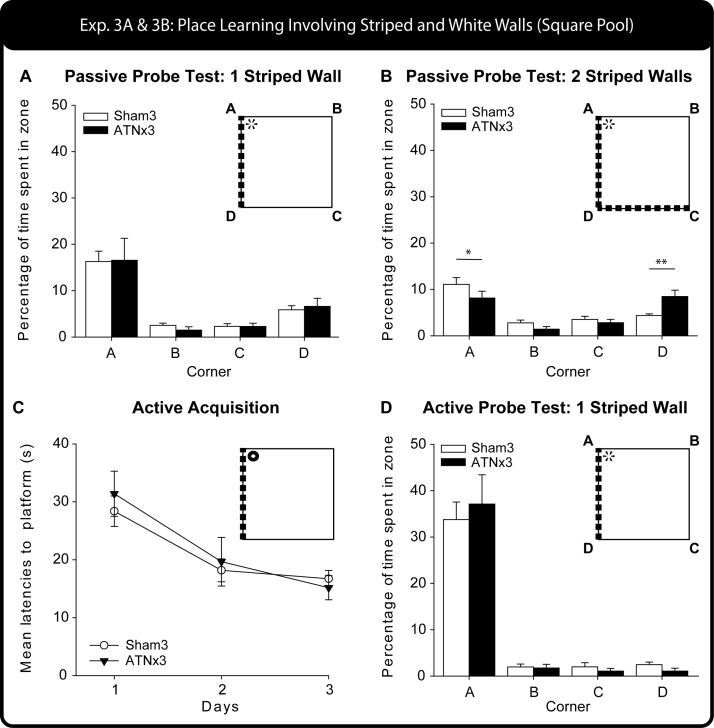
Experiment 3 – Square Pool. The upper graph (A) shows performance on the first Probe Test (60 s), given after passive training with one striped wall and three white walls. The histogram show the percent of all swim time spent in each of the four corners. B) Performance on the second probe trial (60 s) after passive training (see Figure 1D) when the pool had two striped walls and two white walls. The histogram shows the percent of all swim time spent in each of the four corners, * *p* < .05, ** *p* < .01, simple effects group comparison. The lower graphs (C and D) show performance during and after active training, that is, being allowed to swim to the escape location. C) Acquisition performance measured by latency to escape, D) subsequent performance on the Probe Test (60 s) after active training as measured by corner preferences. Data shown are group means, whereas the vertical bars are the standard error of the means (*SEM*). For those probes that were repeated (A and B) the figure shows the combined data.

The times spent in the various corners during the 60-s probes were compared in a three-way mixed model ANOVA [between factor Group (Sham3, ATNx3), within subject factors Zone (correct: A, incorrect: D, white-white near: B, white-white far: C) and Probe (Test, Retest)]. None of the statistics involving Probe was significant and so the Test/Retest data are collapsed in the descriptions below (also Figure 8A). There was no main effect of Group (*p* > .1), nor did Group interact significantly with any other factor (lowest *p* = .09). The overall preference the rats had for the correct corner is reflected in the main effect of Zone, *F*(3, 63) = 25.8, *p* < .001.[Fig-anchor fig8]

Finally, the groups did not differ significantly on either their mean swim speeds or on the mean distance traveled during either probe (both, *p* > .1). However, on this measure there was a main effect of Probe indicating that the rats significantly increased both their swim speed and their distance traveled on Retest compared with Test (swim speed, *F*(1, 21) = 11.22, *p* = .003; distance traveled, *F*(1, 21) = 11.21, *p* = .003). The interactions with Group were not significant (both, *p* > .1).

#### Transfer test probe - two striped walls, two white walls

Figure 7 (middle panel) shows the swim paths of a Sham3 and ATNx3 rat during the 60 s of the first of the two-striped wall probes. Only 25% and 38% of the eight ATNx3 rats swam to the correct corner first during the Test and Retest probes, respectively. A binomial test indicated that the ATNx3 group was not different from chance (assuming 25%) on both probes (both, *p* > .1). Similarly, only 27% of the 15 Sham3 rats swam to the correct corner first during the initial Test Probe (not different from chance, *p* > .1), but during Retest, 60% of 15 Sham3 rats swam to the correct corner first, and binomial tests revealed that their performance was significantly above chance (*p* = .004, one-tailed). The two groups did not, however, differ from one another on this choice measure (Fisher’s Exact Probability: *p* > .1 for both Test and Retest).

The mean latencies to the correct corner for the ATNx3 group were 20.3s (*SEM* = 5.8s) for the initial Probe Test, and 22.3s (*SEM* = 8.4s) for the Retest. Although the Sham3 rats had faster mean latencies [Test: 16.1s (SEM = 1.9s); Retest: 14.9s (SEM = 3.9s)], there was no overall main effect of Group, Probe, or Group × Probe interaction (all, *p* > .1). Likewise, the two groups did not differ on their mean distance traveled (*p* > .1) or mean swim speeds (*p* > .1) on the two Probe Tests.

The percent time in the various corners revealed no effects of Probe (all *p* > .1) and so both tests are grouped together (Figure 8B). There was a main effect of Zone, *F*(3, 63) = 22.69, *p* < .001, and a Group × Zone interaction, *F*(3, 63) = 4.54, *p* = .019, Greenhouse-Geisser correction for violation of sphericity). The simple effects revealed that the Sham3 group spent significantly more time in the correct corner compared with the ATNx3 group, *F*(1, 84) = 4.07, *p* = .047. In contrast, the ATNx3 rats spent significantly more time in the Stripe-Stripe corner compared with the Sham3 group, *F*(1, 84) = 8.03, *p* = .006. The two groups did not differ in the percent of time spent in either the incorrect corner or in the white-white corner (for both, *p* > .1).

### Behavioral Findings: Experiment 3B

After the repeated second probe (two striped walls), the animals underwent active training over four days with four trials per day, though on the fourth day the last trial served as a Probe Test.

#### Acquisition

Figure 8C shows the mean latencies of the Sham3 and ATNx3 groups during the three days of active training. A mixed model ANOVA showed how performance improved with training, Session *F*(2, 42) = 20.0, *p* < .001, but there was no effect of Group or Group × Session interaction (both, *p* > .1).

#### Probe test

Swim paths of individual Sham3 and ATNx3 rats are shown in Figure 7 (right panel). The times spent in the four corners during the probe revealed strong preferences for the correct corner, *F*(3, 63) = 77.5 *p* < .001; Figure 7 right, but no effect of Group or Group × Zone interaction (both *p* > .1). Of the eight ATNx3 rats, 63% chose the correct corner first (*p* = .36), similar to the 60% of the 15 Sham3 rats (*p* = .30; assuming chance 50%) that also first chose the correct corner. The mean latencies to the correct corner were 7.3 s (*SEM* = 1.5 s) and 10.7 s (*SEM* = 3.2 s) for the ATNx3 and Sham3 groups, respectively, and again the groups did not differ (*p* > .1). Similarly, the Sham3 and ATNx3 rats did not differ on the mean distance traveled or on their mean swim speeds (both *p* > .1).

## Discussion

The present study examined the impact of anterior thalamic lesions on learning the location of a goal as specified either by the geometric properties of the environment (Cheng, 1986; Pearce et al., 2004) or by the relative positions of different walls with distinctive appearances (Gilroy & Pearce, 2014). To ensure the rats learned about the relationship between the goal and the specific spatial cue types, the initial stages of training were “passive.” That is, the rat was repeatedly placed on a submerged platform in the escape location, but not allowed to swim to that same location. In more conventional spatial tasks, the rat swims to find the escape platform, normally guided by the spatial relationships between multiple distal room cues, for example, furniture, windows, wall hangings, and lights (stimulus-stimulus learning) thus, it has been claimed, forming a flexible cognitive map (Morris, 1981; O’Keefe & Nadel, 1978; Tolman, 1948; but see Hamilton et al., 2007). Such active navigation is reliant on the hippocampus (Morris et al., 1982; Eichenbaum et al., 1990), though its effective use also depends on the anterior thalamic nuclei (Dumont et al., 2010; Henry et al., 2004; Warburton et al., 2001). However, because rats are required to swim to the goal, responses made just before reaching the escape platform are reinforced. For this reason, rats could also adopt a more rigid stimulus-response strategy, for example, to navigate in a certain direction relative to a chosen landmark outside the pool (Cartwright & Collett, 1983; Hamilton et al., 2007). Such a strategy has been observed in rats with fornix lesions (Eichenbaum et al., 1990). Acquiring a stimulus–response strategy should, however, be prevented by passive training (as responses are not permitted). Furthermore, passive training should limit the contribution of procedural learning to initial task performance (Bannerman et al., 1995; Cain et al., 2006).

In Experiment 1, rats were passively trained to discriminate between the corners of a rectangular pool. Any rectangle contains two different pairs of corners, as diametrically opposite corners have the same geometric properties (Figure 1A). Anterior thalamic lesions severely impaired the ability to differentiate between the correct and incorrect corners. (The term ‘correct’ is used throughout to refer to the corner that had contained the submerged platform during passive training.) This learning deficit was reflected in the failure of the ATNx1 rats to select preferentially the correct corners on the first swim trial after passive training or to prefer the vicinity of the correct corners over the incorrect corners when allowed to swim around the rectangular pool (Probe Test, Experiment 1). To ensure the rats relied on geometric information, the walls of the rectangular pool were all painted gray and the pool was surrounded by a uniform, opaque curtain. In addition, the pool was rotated after every trial (Horne et al., 2012; Pearce et al., 2004).

The present study builds on the finding that anterior thalamic lesions impair active geometric learning in the rectangular pool, that is, when rats swim to the escape platform on every trial (Aggleton et al., 2009). In that previous study, anterior thalamic lesions not only delayed acquisition, as measured by latency to escape, but also disrupted corner preference in a subsequent probe trial when the escape platform was removed (Aggleton et al., 2009). There is, however, a potential confound in the actively trained version as, rather than learn the overall geometric properties of the pool, a control rat could, for example, learn to swim to the longest wall and then turn right (or left; see Figure 1A). This strategy would take the rat to the correct corner (Pearce et al., 2004). This potential confound was removed by training rats passively in the rectangular pool and not following this with active training.

The present findings not only extend the results from active training in a rectangular pool (Aggleton et al., 2009) but also reinforce similarities with the disruptive effects of hippocampal lesions on related geometric tasks (Goodrich-Hunsaker et al., 2005; Jones et al., 2007; McGregor et al., 2004; Pearce et al., 2004). The implication is that both the hippocampus and anterior thalamic nuclei work conjointly (see Dumont et al., 2010; Henry et al., 2004; Warburton et al., 2001) to support this form of spatial learning. One explanation for a deficit in both the passive and active variants of the geometric task is that anterior thalamic lesions, along with hippocampal lesions (Pearce et al., 2004), disrupt the ability to distinguish relative lengths. Such a deficit could then reflect the links the anterior thalamic nuclei and hippocampus have with parietal cortex (Dumont et al., 2010; Pearce et al., 2004; Save & Poucet, 2000). Within the latter region, the retrosplenial cortex is strategically placed given its dense interconnectivity with the hippocampus and anterior thalamic nuclei, as well as with other parietal areas (van Groen & Wyss, 1992, 2003; Vann et al., 2009).

The notion of an anterior thalamic—parietal—hippocampal involvement in relative length discrimination could, in part, be related to the properties of head direction cells. These cells are found in the anterior thalamic nuclei, retrosplenial cortex, and postsubiculum (Chen et al., 1994; Taube, 1995, 2007). It is known that anterodorsal thalamic head-direction cells can use shape information to signal direction (Clark et al., 2012), though in view of the passive training procedure it becomes important to consider whether locomotor activity is necessary for thalamic head direction firing (Knierim et al., 1995; Taube, 1995). In fact, head direction cells in the anterior dorsal thalamus can encode new stimuli with either active or passive movement (Shinder & Taube, 2011), although active exploration may better aid the formation of stable spatial representations in the hippocampus (Rowland et al., 2011). More direct evidence for the likely involvement of the head direction system in the geometric task comes from the finding that lesions of the lateral mammillary nucleus, also part of the head direction system, produce a transient acquisition deficit on the active version of the same rectangular pool task (Vann, 2011). As the lateral mammillary and the anterodorsal head-direction signals are upstream of the hippocampal formation (Goodridge & Taube, 1997; Taube, 1995, 2007) such a contribution would also help to explain the lesser impact of fornix lesions on this geometric task (Aggleton et al., 2009), as this tract does not contain efferents from these diencephalic nuclei.

Experiments 2 and 3 examined the impact of anterior thalamic lesions on learning locations identified by the spatial disposition of distinctive walls (see Gilroy & Pearce, 2014). Testing with passive placement, which was followed by active training, revealed that anterior thalamic lesions produce relatively specific deficits concentrated on the passive training stages. Between Experiments 1 (rectangular pool) and Experiments 2 and 3 (square pool) there were procedural differences in the pretraining methods. These pretraining differences anticipated the training procedures to be employed in the experiment proper. Thus, in Experiment 1, pretraining only involved passive placement, as did the subsequent training regime. In Experiments 2 and 3, where both passive and active learning were assessed, the pretraining was active so as aid the rats on the subsequent switch during the experiment proper from passive to active training. This change in pretraining does, however, restrict direct comparisons between the rectangular (Experiment 1) and square (Experiments 2, 3) pool results.

In Experiment 2 (alternating black and white walls), the ATNx2 rats failed to select the correct corner first on the initial probe test after passive training. This failure was reflected in their significantly higher latencies to first reach the correct corner. This latency difference in Experiment 2 was then carried over into the very beginning of active training in Experiment 2B (Figure 5C), but rapidly disappeared with repeated swims in the pool. The ATNx3 rats also showed raised latencies to reach the escape platform position on their first probe test after passive training in a square pool with three white walls and one striped wall. On this same probe, the ATNx3 rats were at chance when first selecting between the correct white-striped corner and its mirror-image counterpart, although the majority of the control rats first swam to the correct corner.

A further abnormality in Experiment 3 was the excessive preference shown by the ANTx3 rats for the corner formed by the junction of two striped walls in the final set of probe tests (Figures 1D and 8B). An intriguing explanation for this abnormality is that Sham3 rats identified the location of the goal during placement training on the basis of the spatial relationship between the striped and white walls. The single corner with these properties would then still be preferred above all others during the probe test with two adjacent white walls and two adjacent striped walls (Figure 8B). In contrast, the ATNx3 rats may have been unable to acquire such a complex spatial representation and, instead, identified the correct corner as being at a particular end of the striped wall. Using this information, the lesioned rats would then show an equal preference during the probe test for the correct corner and the corner composed of two striped walls. A different account is that the ATNx3 rats learnt that the corner with stripes is positive, making the corner with two stripes even more attractive.

Despite these lesion-induced changes, both set of rats with anterior thalamic lesions (ANTx2, ANTX3) showed a normal preference for the correct corner once it had been reached in the first probe test after passive training (Figures 5, 7, and 8). Equally striking was the ability of the rats with anterior thalamic lesions to learn successfully the escape location in the square pool when actively trained. It should, however, be noted that the active tasks in Experiments 2 and 3 are soluble by other strategies, for example, swim to the dark (or striped) wall and always turn right (see Figure 1). It should also be noted that this spared performance was found despite the anterior thalamic lesions being almost complete. The effectiveness of the surgeries was confirmed by the very poor performance of the ANTx2 rats on T maze alternation, which was sensitive to proactive interference (see also Law & Smith, 2012).

The pattern that emerges from the square pool task (Experiments 2, 3) is that rats with anterior thalamic lesions can still recognize arrangements of wall stimuli, but have difficulty in first using that information to guide them to the correct corner. Consequently, the clearest deficits are found on the first probe test after passive training. On this test, the rats not only need to have learnt the array of cues that define a particular corner, but must also translate this image to make a match when first viewed from a novel location (the middle of the pool). In more standard swimming tasks, a potentially similar dissociation between how to first get there (impaired) and recognizing the correct location once reached (spared) has been described in rats with an isolated hippocampal CA1 field (Brun et al., 2002) and in rats with fimbria-fornix lesions (Eichenbaum et al., 1990; Whishaw et al., 1995). Such studies have also shown how flexible cue use depends on the fornix (Eichenbaum et al., 1990). Other pertinent evidence shows that the anterior thalamic nuclei are important for orienting and heading toward specific landmarks, that is, ‘getting there’ (Calton et al., 2003; Taube, 2007; Wilton et al., 2001), while the ability to translate and change spatial frames of reference has been linked to retrosplenial cortex function (Burgess, 2002; Byrne et al., 2007; Vann et al., 2009). Furthermore, a number of studies have shown that anterior thalamic lesions produce retrosplenial cortex dysfunctions (Dumont et al., 2012; Garden et al., 2009; Jenkins et al., 2004; Sutherland & Hoesing, 1993). The overall implication is that in the intact brain the hippocampus, anterior thalamic nuclei and retrosplenial cortex would normally work together to enable the spatial translations required for the rat to navigate effectively on the first probe test after passive training, but that this cooperation is impaired when the anterior thalamic nuclei and hippocampus are disconnected, for example, by fornix lesions (Eichenbaum et al., 1990; Whishaw et al., 1995). At the same time, anterior thalamic lesions also impair spatial tasks with little or no navigational component (Dumont et al., 2014; Wilton et al., 2001), showing that this loss in ‘getting there’ is not a sufficient account for the lesion effects on spatial learning.

The two different explanations (length discrimination and spatial cue translation for navigation) for the respective deficits in the rectangular and square pool tasks are consistent with other evidence that these thalamic nuclei have multiple spatial functions, that is, their involvement in spatial learning is appreciably more than just providing head direction information from the anterodorsal nucleus. Supporting evidence includes the finding that selective lesions within the anterior thalamic nuclei that spare the anterodorsal nucleus can still disrupt spatial learning (Aggleton et al., 1996; Byatt & Dalrymple-Alford, 1996). Likewise, selective lesions within the anterior thalamic nuclei that target the anterodorsal nucleus, but spare the anteromedial nucleus, have less impact than complete anterior thalamic lesions (Aggleton et al., 1996; Byatt & Dalrymple-Alford, 1996; van Groen et al., 2002). This latter result accords with the finding that lesions of the lateral mammillary nucleus, which abolish the anterodorsal head direction signal (Blair et al., 1998, 1999), produce only transient deficits on spatial tasks (Vann, 2005, 2010, 2011). Electrophysiological and anatomical studies also reveal the likely involvement all three major anterior thalamic nuclei in different aspects of spatial processing (Aggleton et al., 2010; Albo et al., 2003; Tsanov et al., 2011; Vertes et al., 2001, 2004; Wright et al., 2013).

The present findings help to reveal the multiplicity of spatial functions served by the anterior thalamic nuclei. At first sight, the spared abilities shown by the ATNx2 and ATNx3 rats on the active conditions in the square pool (Experiments 2, 3) might seem surprising given how poorly rats with anterior thalamic lesions perform on standard (active) watermaze procedures, where probe test data show how the animals seemingly fail to recognize the platform locations, even when it is reached (van Groen et al., 2002; Warburton et al., 1999, 2001). It is likely, however, that the selectivity of the present deficits in the square pool reflects the unusually controlled environment employed in the current study, in which the spatial cues (pool walls) were highly salient and limited in number. In contrast, the greater severity of the anterior thalamic lesion deficits in standard watermaze tasks presumably reflects the increased complexity of the problem arising from the involvement of multiple aspects of learning, the heterogeneous array of distal spatial cues, and the likely importance of judgments about relative distance (Bannerman et al., 1995; Hamilton et al., 2007; Sutherland & Rodriguez, 1989).
